# An Input-Perceptual Reconstruction Adversarial Network for Paired Image-to-Image Conversion

**DOI:** 10.3390/s20154161

**Published:** 2020-07-27

**Authors:** Aamir Khan, Weidong Jin, Muqeet Ahmad, Rizwan Ali Naqvi, Desheng Wang

**Affiliations:** 1 School of Electrical Engineering, Southwest Jiaotong University, Chengdu 611756, China; aamir@my.swjtu.edu.cn (A.K.); wds@my.swjtu.edu.cn (D.W.); 2 China-ASEAN International Joint Laboratory of Integrated Transport, Nanning University, Nanning 530000, China; 3 School of Information Science and Technology, Southwest Jiaotong University, Chengdu 611756, China; muqeetahmad@my.swjtu.edu.cn; 4 Department of Unmanned Vehicle Engineering, Sejong University, Seoul 05006, Korea; rizwanali@sejong.ac.kr

**Keywords:** image-to-image conversion, image de-raining, label to photos, edges to photos, generative adversarial network (GAN)

## Abstract

Image-to-image conversion based on deep learning techniques is a topic of interest in the fields of robotics and computer vision. A series of typical tasks, such as applying semantic labels to building photos, edges to photos, and raining to de-raining, can be seen as paired image-to-image conversion problems. In such problems, the image generation network learns from the information in the form of input images. The input images and the corresponding targeted images must share the same basic structure to perfectly generate target-oriented output images. However, the shared basic structure between paired images is not as ideal as assumed, which can significantly affect the output of the generating model. Therefore, we propose a novel Input-Perceptual and Reconstruction Adversarial Network (IP-RAN) as an all-purpose framework for imperfect paired image-to-image conversion problems. We demonstrate, through the experimental results, that our IP-RAN method significantly outperforms the current state-of-the-art techniques.

## 1. Introduction

The main objectives of image-to-image conversion tasks are the discovery of suitable latent space and understanding of features maps from source to target images. These tasks have multiple applications in computer graphics, image processing, and computer vision. Image processing applications include: (i) image in-painting, where damaged parts of an image are restored [1,2], (ii) image de-raining where rain-streaks are removed from an input image to get rain-free image [3,4], (iii) image super-resolution where high-quality images are generated from similar degraded images [5,6,7,8,9,10]. Additional applications exist, however they are not constrained to image denoising [11,12,13], style transfer [14], image segmentation [15] and image colorization [16,17].

Recently, researchers have developed convolutional neural networks (CNNs) for multiple image-to-image conversion problems. These models mostly come in the form of an encoder-decoder structure where the encoder encodes an input image to some latent space, the decoder decodes from the latent space to the required output image and then they punish the network with a loss function to pick up the mapping between two image domains. Many different loss functions and distinct motivations [5,18] established these models. CNNs utilize reconstruction or pixel-wise losses [5,17,19,20] to generate output images, which are the most upfront techniques. For example, in pixel space, the least absolute or the least-squares losses used to estimate the difference between the ground-truth and generated images. Pixel-wise computation can construct sensible photos. However, in many cases, these losses just capture low-frequency instead of high-frequency components of images, leading to some critical flaws concerning the outputs, e.g., image blurring and image artifacts [7].

Recent years have witnessed that the procedures using the concept of generative adversarial networks (GANs) [21] have accomplished remarkable results in image-to-image conversion tasks. GANs, introduced by Goodfellow et al., is made up of a generator network G and a discriminator network D, targeting to model the real images distribution by synthesizing generated samples, which are very similar to real images. GAN-based models need more memory and computational time in the training process than simple CNN based models as they need to train two networks, i.e., the Discriminator network and the Generator network [22]. Whereas in the testing process, there is only one network, i.e., the generator network. Therefore, the memory and the computational time of GAN based models in the testing process are nearly similar to CNN based models. The significant advantage of using GAN based model is that it generates sharper and more realistic images than CNN based models [23,24,25]. Hence, the algorithms using the concepts of GANs and conditional GANs (cGANs) [26] have turned out to be a common approach for numerous image-to-image conversion tasks [8,23]. Based on cGAN, pix2pix-cGAN [23] became a representative method aimed at solving the paired image-to-image conversion problems, the objective of which is to map the conditional distribution of the real images conditioned on the given input images [25,27,28,29].

**Figure 1:**
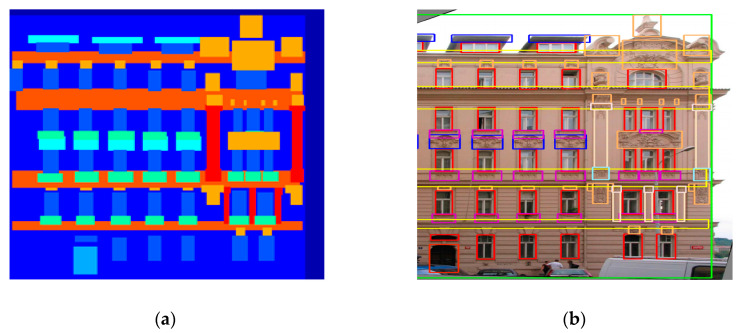
Example of the label to architectural photos. (**a**) shows an input labeled image. (**b**) shows marked objects in a ground-truth image.

The critical part of image-to-image conversion tasks is that they have to map high-resolution input grids into high-resolution output grids. Additionally, the issue we consider is that the input and the output have dissimilar surface nature, but both must render the same basic structure to ensure perfect outcomes. There are two popular methods to find out the basic structure of an image, i.e., perceptual features based method [6] and moments based method [30]. The key challenges with methods of moments (MoM) [31] for training deep generative networks are in describing millions of sufficient distinct moments and identiflying an objective function for learning the desirable moments [31,32]. On the other hand, the use of features from deep neural networks (VGG-16) pre-trained on ImageNet dataset [33,34] has led to important advancements in computer vision. Perceptual features have been widely used in piece of works such as super-resolution [6], style transfer [14], and transfer learning [35]. The image generation model is considered to learn from the information in the form of input images, which plays a significant part in the image-to-image conversion task to achieve desire targeted outputs. In paired image datasets, the input structure is roughly matched with the output structure and can significantly affect the production of the image generation models. For example, Figure 1 shows that the window frames are not accurately labeled in the corresponding input images. Hence, the image generation model requires further information to capture targeted high-resolution output grids against each given missing high-resolution input grids. Despite considerable progress, we note that the previous approaches have not examined optimized additional input information for imperfect paired datasets. 

To overcome the problem of imperfect paired datasets and to attain desired results, we opted to feed this extra information in the form of input-perceptual loss (i.e., calculated between imperfect paired images) into the objective function of the proposed model. It is an essential issue, as the perfect paired dataset is expensive and hard to collect. This work introduces a trade-off between collecting large-amount of the perfect paired dataset and an optimized training for the image-to-image conversion network.

The remainder of the study is as follows: We discuss the previous research of the image-to-image conversion with details in Section 2. The IP-RAN methodology, objective, and network architecture are explained in Section 3. In Section 4, we present the experiments, results, and analysis of different loss functions and generator configurations. Section 5 presents the conclusions and future work.

## 2. Related Work

In previous years, the training of deep convolutional neural networks using back-propagation algorithms with per-pixel loss functions has solved a broad range of feed-forward image-to-image conversion tasks [18,36]. Various techniques of image-to-image conversion employ only pixel-level losses or pixel-level losses preceded by several additional losses [6,23]. Image segmentation techniques generate dense scene labels by operating networks in a fully convolutional way over a single input image [20,37,38,39,40]. Image de-raining techniques try to eliminate rain strikes in uncontrolled weather images [41,42]. Image super-resolution techniques generate a high-resolution image for a given its low-resolution matching part [5,6]. Image in-painting is designed to retrieve the missing portion of the given damaged image [1,43,44]. Other examples of image-to-image conversion techniques modeled on feed-forward CNNs exist, however, they are not constrained to depth estimations [37,45,46] and image colorization [19], etc.

A series of GAN-family [16,26,47,48] networks was introduced in a short time for an enormous variety of problems since Goodfellow introduced the influential concept of Generative Adversarial Nets (GAN) [21] in 2014. GANs also showed promising results in numerous applications for computer vision, for example, image generation, representation learning [48], image editing [49], etc. Specifically, various extended GANs accomplished good results at several image generation applications such as style transfer [24], super-resolution [7], image inpainting [1], text2image [50], and like many other domains including videos [51] and 3D data [52]. These studies also consist of but are not constrained to the PGN introduced for video prediction [53], the iGAN introduced for interactive application [54], the SRGAN added for super-resolution [7], and the ID-CGAN presented for image de-raining [3].

Moreover, some of these works based on GANs are dedicated to developing an improved generative model, for example, WGAN(-GP) [55,56], Energy-based GAN [57], Progressive GAN [58], SN-GAN [59] and E-GAN [60]. A conditional image generation based on GANs has also been actively studied recently. Some advanced GAN models continuously improved the quality of particular tasks, e.g., InfoGAN [16], cGANs [26], and LAPGAN [61] have been introduced to image translation recently for their easy execution and outstanding results. The cGANs [26] hold category labels as conditional data for the generation of particular images. Some of the works have included GANs into their designs to enhance the efficiency of conventional tasks, e.g., for small entity (or object) detection, the PGAN [62] was adopted. Specifically, Li et al. [62] developed an innovative perceptual-discriminator network, which includes a perception block and an adversarial block. Wang et al. [25] used different layers of discriminator network to measures perceptual losses. Sung et al. [63] introduced new paired input conditions for the replacement of conditional adversarial networks to improve the image-to-image translation tasks.

Additionally, some modifications of the GANs [29,64,65,66] examined cross-domain image conversions over discovering the linear mapping relationship among various image domains. In particular, primal GAN intentions to investigate the mapping relationships between input images and target images, although a double (or opposite) GAN does the opposite task. Such GANs shape a closed-loop and enable the translation and reconstruction of images from either domain. These designs can also be used to execute image conversion operations in the lack of paired examples by merging cycle consistency loss and GAN loss. However, paired data is available for training in specific applications, Ge et al. [29], Zhu et al. [64], Yi et al. [65], and Kim et al. [66] ignore that paired data often achieves less than paired methods [23]. It is therefore still essential at this point to study paired data training, particularly for performance motivated circumstances and implementations like the photo-realistic picture synthesis [7], high-resolution image synthesis [8], real-world image painting [67], etc. 

In GANs based works, generator networks are the same as the aforementioned encoder-decoder structure in CNNs. As the training of deep CNNs suffer from vanishing gradient problem. Therefore, many previous works [3,4,25] used skip-connections in the generator to pass the gradient easily to prior layers of the encoder. Unfortunately, these skip-connections directly carry unwanted information from the inputs to the resultant images, hence affecting the visual quality of the constructing images. In the demand to develop a visually appealing image-to-image conversion model, we have to consider the following facts into the optimization method:

- The principle, to perfectly map targeted output images must not be affected by the texture of the given input images, which should be the essential pillar in the formation of a generator structure.
- The visual quality of constructed images should also be considered in the optimization method rather than just relying on qualitative performance metric values. This principle can guarantee that the generated images look visually appealing and realistic.

Under the above criteria, we present the Input-Perceptual and Reconstruction Adversarial Networks (IP-RAN) for image-to-image conversion tasks. The IP-RAN consists of an encoder-decoder network G; for converting an input image to the desired output image, a discriminator network D; to flag the real or fake photos and an input-perceptual loss network P; to calculate fundamental structure difference between an input image and the ground-truth image. We employe the input-perceptual, the traditional reconstruction L1, and the generative adversarial losses in the objective function. Initially, this work utilized the input-perceptual loss to calculate the missing information of the basic structure in the input images according to the target images. Then, this study used similar to many traditional losses the L1 loss for penalizing generated images to be near to the targeted images. Meanwhile, we used the generative adversarial losses to estimate the distribution of converted images, i.e., to punish the generated distribution for converging into the target distribution of output, which generally results in the production of more visually pleasing images. The contributions of this study are as follows:

- This study introduces a novel approach to deal with imperfect paired datasets and the method of feeding extra information into the objective function in the form of input-perceptual losses calculated between the input images and the target images for imperfect paired datasets.
- We introduce an optimized method based on pix2pix-cGAN and conditional GANs (cGANs) frameworks for existing imperfect pair datasets.
- We also analyzed the primary two different configurations of the generator structure, and the results show the proposed approach is better than previous methods.
- We achieve both qualitative and quantitative results by using IP-RAN, which indicates that the adopted technique produces better results than the baseline models.

Table 1 shows a comparison between the proposed and existing methods.

## 3. Methodology

In this work, we have two sets of paired training images, i.e., a set of input images ${\left\{x_{i}\right\}}_{i=1}^{N}\in X$ and a set of target output images ${\left\{y_{i}\right\}}_{i=1}^{N}\in Y$. We train the generative network G that the fake generated images G(x) to be same as the real targeted images, and alongside we train a discriminative network D to distinguish the fake generated images G(x) from the real targeted images. The generator network learns the mapping from an input domain to a real-world domain by minimizing adversarial losses, aiming to deceive the discriminator network. The generator has sub-networks: an encoder Enc, residual blocks Res, and a decoder network Dec. The encoder network contains a sequence of convolutional layers, which convert an input image into encoded feature space Enc(x). Later, the output of encoder network, Enc(x), becomes the input of residual blocks [68]. The output of the residual layers, Res(Enc(x)), is the activation maps which feed to the decoder network Dec. At that moment, a sequence of fractionally-stride convolutionary layers decode the converted features into the fake generated image G(x). Equation (1) expresses the output of the generator network:

(1) G(x)=Dec(Res(Enc(x)))

**Figure 2:**
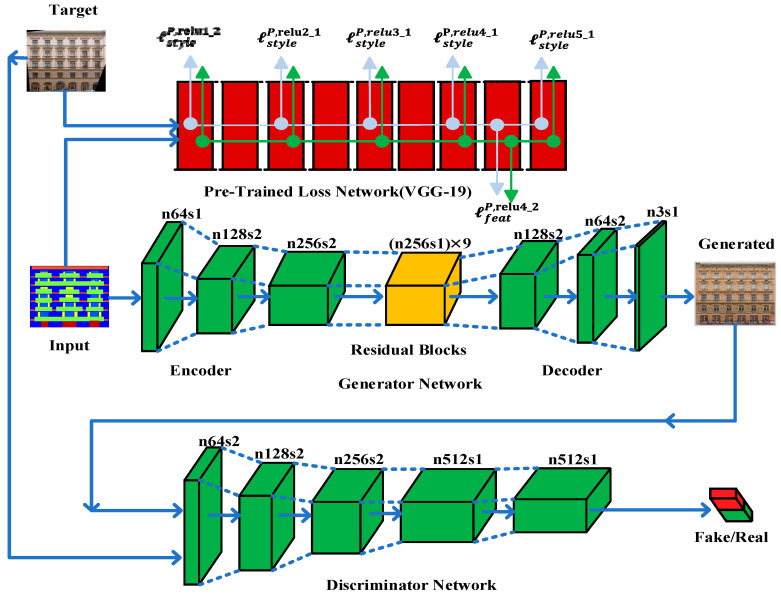
IP-RAN framework. IP-RAN consists of generator network, G, input-perceptual loss network, P, and discriminator network, D. The generator network, G, is intended to generate translated images from given input images. It is composed of an encoder-decoder structure that includes two down-sampling layers of stride-2 convolution, several residual blocks, and two up-sampling layers stride-2 of transposed convolution. Input-perceptual loss network, P, is the pre-trained VGG-19 and used to extract features from hidden layers to calculate the perceptual loss. The discriminator network, D, consists of convolutional-BatchNorm-LeakyRelu layers, and its output is used to distinguish generated images from real images.

The whole network architecture is shown in Figure 2 and is called the Input-Perceptual Pixel-Reconstruction Generative Adversarial Networks (IP-RAN).

### 3.1. Objective

The input-perceptual loss calculated between high-resolution input grids and targeted high-resolution output grids, which decrease the effect of less information in the input images and useful against imperfect paired datasets. Equation (2) expresses input-perceptual loss:

(2) $${ℒ}_{P}\left(P\right)=\varphi_{c}l_{f}+\varphi_{s}l_{s}$$

where $l_{f}$ is the feature reconstruction as given in Equation (3), and $l_{s}$ is the style reconstruction losses as given in Equation (4), are the two parts of the perceptual loss function, as Johnson et al. described in [6]. Input-perceptual losses are utilized to measure fundamental structural differences such as common patterns, texture, colors, etc., between the high-resolution input grids and the high-resolution target grids. 

Let $P_{i}\left(x\right)$ be the activation maps for the $i^{th}$ layer of the network P when processing the image x. If i is a convolutional layer then $P_{i}\left(x\right)$ will be an activation map having a shape of $C_{i}\times H_{i}\times W_{i}$. The feature reconstruction loss can be calculated as Euclidean distance between activation maps as follows:

(3) $$l_{f}=\ell_{feat}^{P,i}\left(x,y\right)=\frac{1}{C_{i}H_{i}W_{i}}\;||P_{i}\left(x\right)-P_{i}{\left(y)|\right|}_{2}^{2}$$

where $P_{i}$ denotes the non-linear CNN transformation at the $i^{th}$ layers of the loss network, P. The $\ell_{feat}^{P}$ loss aims to measure the discrepancy between high-level features of the given images. 

The style reconstruction loss can be computed as squared Frobenius norm for the discrepancy between the Gram matrices of the input and the targeted images as follows:

(4) $$l_{s}=\ell_{style}^{P,i}\left(x,y\right)=\;||G_{i}^{P}\left(x\right)-G_{i}^{P}{\left(y)|\right|}_{F}^{2}$$

where $G_{i}^{P}\left(x\right)$ is the Gram matrix of $i^{th}$ layer activation maps of a given image x extracted from network P. $G_{i}^{P}\left(x\right)$ is defined as the components of the $C_{i}\times C_{i}$ matrix is given by:

(5) $$G_{i}^{P}{\left(x\right)}_{c,c^{*}}=\frac{1}{C_{i}H_{i}W_{i}}\overset{H_{i}}{\underset{h=1}{\sum }}\overset{W_{i}}{\underset{w=1}{\sum }}P_{i}{\left(x\right)}_{h,w,c}P_{i}{\left(x\right)}_{h,w,c^{*}}$$

where $P_{i}\left(x\right)$ interpret as giving $C_{i}$-dimensional activation maps for each point on $H_{i}\times W_{i}$ grid, and the Gram matrix, $G_{i}^{P}\left(x\right)$, relates to non-centric covariance of the $C_{i}$-dimensional activation maps, processing each grid site as an autonomous sample. Therefore, it gathers details about the features that appear to be working together. The Gram matrix can also be determined accurately by transforming $P_{i}\left(x\right)$ into a matrix ϕ of shape $C_{i}\times H_{i}W_{i}$; then GiP(x)= ϕϕTCiHiWi. 

Generative adversarial loss [21], which trains G and D together as the two-player mini-max game with loss function ${ℒ}_{GAN}\left(G,D\right)$. The generator network G attempts to produce an image G(x) that appears similar to the image in the target domain Y, while the discriminator network D attempts to differentiate between them. In particular, we train the discriminator network, D, to maximize the likelihood of classifying the correct label to the targeted image and the generated image G(x), while training G is to minimize the likelihood of classifying the correct label to the generated image G(x). The mini-max game can be formulated as:

(6) $$\underset{G}{\min}\;\underset{D}{\max}\;E_{y\in Y}\left[\log\left(D\left(y\right)\right)\right]+E_{x\in X}\left[\log\left(1-D\left(G\left(x\right)\right)\right)\right]$$

GANs-based models have revealed the significant ability to learn generative models, particularly for image generation tasks [16,53,55]. Therefore, we also implement the GANs learning process to resolve image conversion tasks. As illustrated in Figure 2, the image generation network G is used to produce output image, G(x), against the input image, x∈X. In the meantime, each input image $x_{i}$ has a correspondent target image $y_{i}$. We assume that all target images, y, follow the distribution y∈Y, and the generated images, G(x), are motivated to have matching distribution as targeted images y, i.e., G(x)~Y. Besides, to accomplish the generative adversarial learning approach, a discriminative network, D, is added, and the adversarial loss function can be expressed as follows:

(7) $$\underset{G}{\min}\;\underset{D}{\max}\;V\left(G,D\right)=E_{y\in Y}\left[\log\left(D\left(y\right)\right)\right]+E_{x\in X}\left[\log\left(1-D\left(G\left(x\right)\right)\right)\right]$$

We use least squares loss (LSGAN) as discussed in [69], which offers a non-saturated and smooth gradient for discriminator network D. Adversarial loss, ${ℒ}_{GAN}\left(G,D\right)$, is expressed as:

(8) $${ℒ}_{GAN}\left(G,D\right)=E_{y\in Y}\;\left[{\left(D\left(y\right)-1\right)}^{2}\right]+E_{x\in X}\left[D{\left(G\left(x\right)\right)}^{2}\right]$$

The generative adversarial loss turns as per the numerical measurement to punish the variance between the distributions of generated images and ground-truth images.

**Figure 3:**
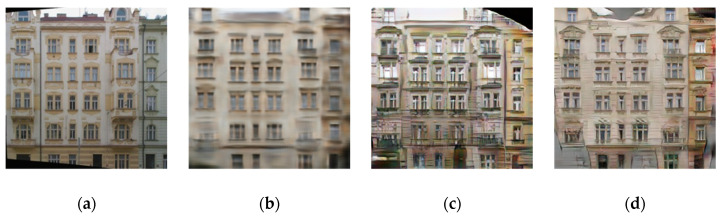
(**a**) ground-truth image, (**b**) image generated by conventional CNN using L1 loss function, (**c**) image generated by standard GAN using adversarial loss and (**d**) image constructed by the proposed method with Input-Perceptual and Reconstruction Adversarial losses

The basic GAN framework is unstable as it trains two competing neural networks. In [64], the author noted that one cause for instability is that there are un-unique solutions during the training of the generator. As shown in Figure 3, several artifacts introduced by the standard GAN structure can be observed which significantly impacts the visual quality of the output image. Previous methods have found that it is useful to combine GAN objectives with more traditional losses such as L2 loss [1] in such way that the work of the discriminator remains unchanged as in Equation (8), but the task of the generator is not only to deceive the discriminator but also to make generated image closer to the targeted ground truth image according to L2. In our method, we used L1 distance instead of L2, because L1 encourages blur reduction:

(9) $${ℒ}_{L1}\left(G\right)=E_{x,y}\left[||y-G{\left(x)|\right|}_{1}\right]$$

The adversarial loss helps the generator and protect from the blurry effect of L1 loss as well as remain close to the targeted output images. The final objective for the generator network is expressed as:

(10) $${ℒ}_{G_{T}}=\varphi_{g}{ℒ}_{GAN}\left(G\right)+\varphi_{L1}{ℒ}_{L1}\left(G\right)+\varphi_{P}{ℒ}_{P}\left(P\right)$$

where ${ℒ}_{G_{T}}$ represents the total generator network loss which is the sum of the generator’s adversarial loss, ${ℒ}_{GAN}\left(G\right)$, L1 reconstruction loss, ${ℒ}_{L1}\left(G\right)$, and the input-perceptual, ${ℒ}_{P}\left(P\right)$.

### 3.2. Network Architecture

Figure 2 demonstrates the proposed structure consisting of three CNNs networks, i.e., the generator network, G, the input-perceptual loss network, P, and the discriminative network, D.

Recently, many solutions [3,23,25] to these problems used skip-connections in the generator network to shuttle the information directly from input to output throughout the network and to solve the vanishing gradient problem. On the one hand, skip-connections are useful in resolving the vanishing gradient problem. Still, for image-to-image conversion problems, these skip-connections are carrying unwanted information from the input throughout the network and influencing the performance of the results critically, see Figure 2. We utilize the ResNet [68] framework same as Johnson et al. [6], with an encoder-decoder structure instead of skip-connections between encoder-decoder layers to avoid unwanted information coming from the input and to produce visually pleasing results. Our generator network includes two downsampling layers of stride-2 convolution, nine residual blocks, and two upsampling layers with stride-2 of transposed convolution and utilizes instance normalization [70], for specifications, see Table 2. The input-perceptual loss network, P, uses VGG-19 pre-trained on the ImageNet dataset [33,34]. We extract features from six layers (Relu-1 of block1, Relu-1 of block2, Relu-1 of block3, Relu-1 of block4, Relu-1 of block5) for style loss $l_{s}$ and Relu-2 of block4 for feature loss $l_{f}$ of pre-trained VGG-19 to calculate input-perceptual losses.

In this work, we use 70 × 70 Markovian PatchGANs [7,23,71] for the discriminator network D to classify whether 70 × 70 overlapping patches of images are real or fake. Patch-level discriminator has fewer parameters than a full-image discriminator and can operate in a fully convolutionary fashion on images of arbitrary size [23].

## 4. Experiments and Results

In this section, we first discuss the specifications of the datasets, proposed model, and training parameters. We compared the IP-RAN with the standard approaches and current state-of-the-art methods. We also discuss the information on the experiments and performance measures used to test the proposed method.

### 4.1. Datasets

Experiments are carried out on several datasets to evaluate the performance of IP-RAN and other state-of-the-art methods. We use three public paired datasets which are as follows:

- CMP facades dataset [72] is used to train for architectural “Labels to Photos” task.
- Dataset provided by ID-CGAN [3] is used to train for the “Image De-raining” task.
- Dataset formed by pix2pix [23] is used to train for the “Edges to Photos” task. The original dataset has come from [54] and [73], and the use of the HED edge detector [74] to extract edges. All images are scaled to 256 × 256.

### 4.2. Model and Parameter Details

In this subsection, we discuss the model and the parameter details. In the case of GAN loss (${ℒ}_{GAN}$), we replace the criterion of negative log-likelihood with a least-square loss [69] for the network’s training stabilization. This least-square loss is found more stable throughout the training procedure and produces higher quality results. In general, for ${ℒ}_{GAN}\left(G,D\right)$, we set that G, train to minimize $E_{x\sim p_{data}\left(x\right)}\left[{\left(D\left(G\left(x\right)\right)-1\right)}^{2}\right]$ and D, train to minimize $E_{y\sim p_{data}\left(y\right)}\left[{\left(D\left(y\right)-1\right)}^{2}\right]+E_{x\sim p_{data}\left(x\right)}\left[(D{\left(G\left(x\right)\right)}^{2}\right]$. Furthermore, we divide the discriminator’s criterion by 2 when optimizing D, which slows the learning rate of D proportional to G. We apply the Adam optimizer [75] and use minibatch Stochastic Gradient Decent (SGD), setting a learning rate of α=0.0002, β1=0.5. Relu activation function, with slope value of 0.2, is used in the generator network, G, except the last layer used tanh. The Batch size is set to one for all of the experiments. The training parameters are set as $\varphi_{g}=1$, $\varphi_{L1}=10$, $\varphi_{s}=1$ and $\varphi_{c}=0$ for labels to photos task,$\;\varphi_{g}=1$, $\varphi_{L1}=10$, $\varphi_{s}=1$ and $\varphi_{c}=1\times 10^{-6}$ for edges to photos task, and $\varphi_{g}=1\times 10^{-9}$, $\varphi_{L1}=10$, $\varphi_{s}=1$ and $\varphi_{c}=1\times 10^{-6}$ for image de-raining task. 

### 4.3. Evaluation Criteria

For a performance demonstration of image-to-image conversion tasks, we performed qualitative and quantitative tests to determine the quality of the generated images. We directly present input and generated images for qualitative assessments. We apply quantitative measures on test sets to assess the performance of different model and configurations such as, Peak Signal to Noise Ratio (PSNR), Structural Similarity Index (SSIM) [76], Visual Information Fidelity (VIF) [77], and Universal Quality Index (UQI) [78]. These quantitative measures valuation are based on the luminance channel of the image. FID score [79] determines the distance between the real data distribution and the generated data distribution.

### 4.4. Analysis of Different Loss Functions

**Figure 4:**
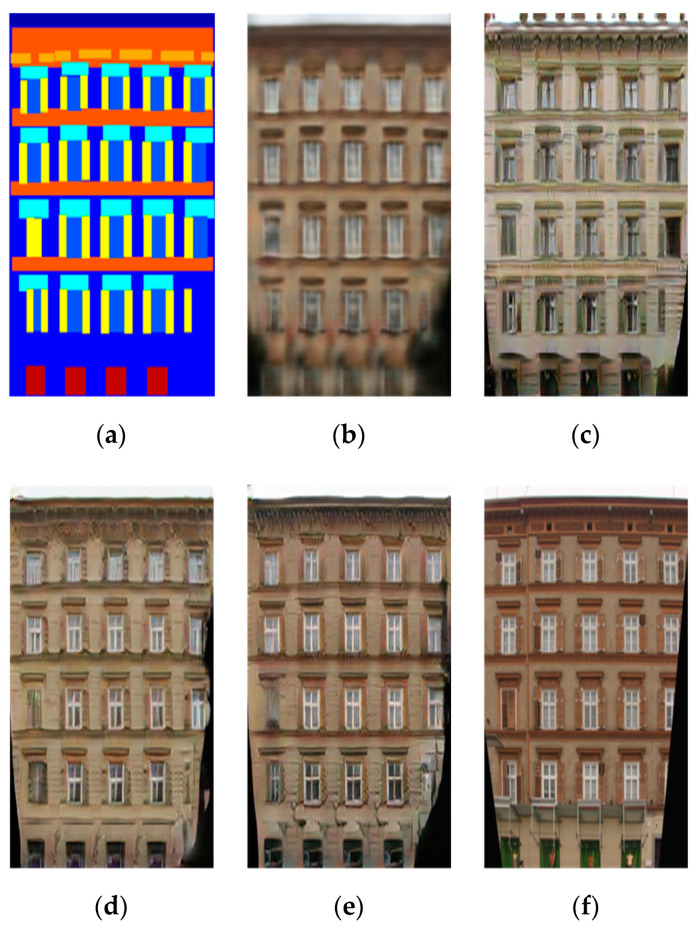
Shows label input against different loss functions that produce different architectural photo results. (**a**) input label image, (**b**) result of L1 (${ℒ}_{L1}\left(G\right)$) alone, (**c**) result of cGAN (${ℒ}_{GAN}\left(G\right)$ ) alone, (**d**) result of L1+cGAN (${ℒ}_{GAN}\left(G\right)+{ℒ}_{L1}\left(G\right)$), (**e**) result of the IP-RAN, and (**f**) target output photo.

We train models to separate the effect of different variations of loss functions on the architectural CMP facades “label to photos” dataset. We perform tests to compare the impact of each part of Equation (10). Figure 4 shows the qualitative results of the variations mentioned below on labels to photos problem.

- L1, by setting $\varphi_{g}=0$ and $\varphi_{P}=0$ in Equation (10), causes to generate blurry outputs.
- The cGAN, by setting $\varphi_{L1}=0$ and $\varphi_{P}=0$ in Equation (10), leads to much sharper outputs but brings visual artifacts.
- L1 and cGAN together, by setting $\varphi_{P}=0$ in Equation (10) causes sensible results but still far from the targeted outputs.
- The results of the proposed loss function in Equation (10), show a significant improvement in quality and similarity to the targeted results.

In Table 3, we compared the abovementioned cases quantitatively using the PSNR, SSIM, UQI, VIF, and FID scores on the labels to photos dataset. L1 achieves higher scores in PSNR, SSIM, UQI, and VIF, but the output results are blurred images and are very poor in FID-score. Hence, pointing out that the results are visually unpleasant. We observed from Figure 4 and Table 3 that for blurry images PSNR, SSIM, UQI, and VIF evaluation scores perform inferiorly. Table 3 shows that cGAN alone achieves poor scores in PSNR, SSIM, UQI, and VIF, which indicating that results are less similar to the targeted output. However, it has got a good FID-score as compare to L1 that shows results have a recognizable structure. Table 3 shows that the IP-RAN achieves the best possible scores in PSNR, SSIM, UQI, VIF, and FID. Hence, the results are similar to the targeted output as well as have a recognizable structure, and they are visually pleasing.

### 4.5. Analysis of Different Generator Configuration

**Figure 5:**
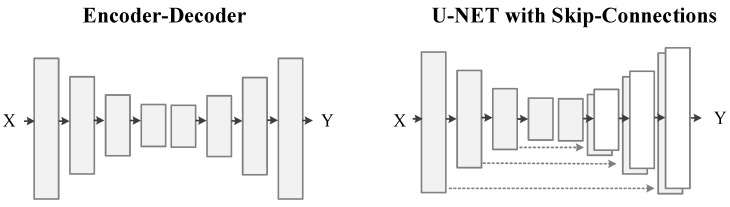
Different structures of image-to-image generation networks.

The encoder-decoder structure does not have skip-connections among the layers. The U-Net structure has skip-connections between encoder layers and decoder layers, as shown in Figure 5. We have trained both structures on image de-raining dataset and labels to photos dataset with similar loss function using pix2pix-cGANs [23] architecture. We conducted tests to compare both structures. 

**Figure 6:**
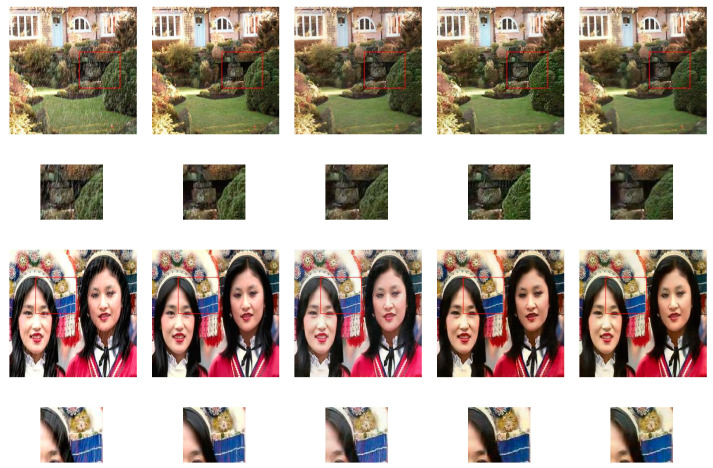

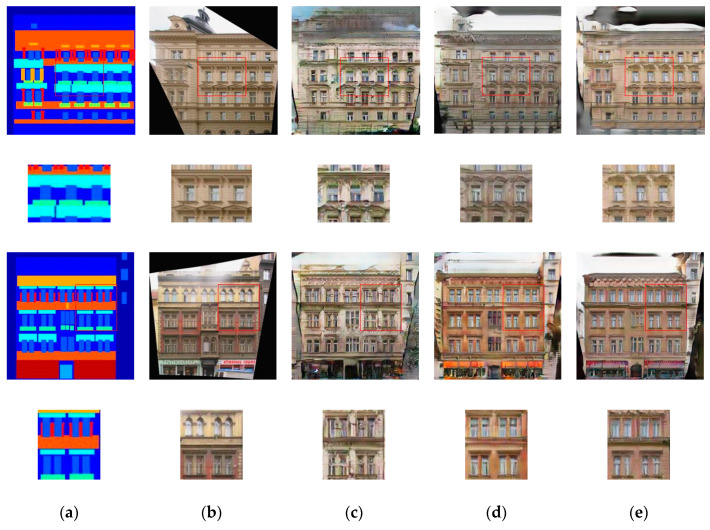
Sample results in the first four rows of rainy to de-raining images and last four rows of labels to architectural photos. For good visual comparison, the smaller images below the test images represent specific regions-of-interest. (**a**) input images, (**b**) targeted photos, (**c**) U-Net with skip-connections, (**d**) Encoder-Decoder and (**e**) IP-RAN.

Figure 6 shows the encoder-decoder structure achieves excellent results without losing any information than the U-Net structure. Skip-connections passing unwanted information of the input images, which have a severe influence on generated images, leads to corrupted results and poorly achieved their targets. In the image de-raining task, the generator structure with skip-connections poorly converts between the rain to de-raining images. In Figure 6c the first four rows, where rain-streaks still can be found in resultant images. The resultant images inherit this unwanted information via skip-connections from the corresponding input images. Figure 6c the last four rows, where resultant images contain bluish and greenish color effects, which are directly coming from the input labeled images via skip-connections.

### 4.6. Comparison with Baseline

For comparison purposes, we selected the following latest state-of-the-art approaches for image-to-image conversion problems:

- Pix2Pix-cGAN [23]: Pix2pix is designed for paired image datasets based on the cGAN architecture. Pix2Pix utilizes L1 reconstruction loss and adversarial loss to train its model for the conversion of input images to output images.
- UTN-GAN [29]: UTN-GAN introduced a GAN-based unsupervised transformation network with hierarchical representations learning and weight-sharing technique. The reconstruction network learns the hierarchical representations of the input image, and the mutual high-level representations are shared with the translation network to realize the target-domain oriented image translation.
- PAN [25]: PAN can learn a mapping function to transform input images to targeted output images. PAN consists of a image transformer network and a discriminator network. In PAN, the discriminator measures perceptual losses on different layers and identifies between real and fake images. PAN uses perceptual adversarial losses to train the generator model.
- iPANs [63]: iPANs used U-NET as image transformation network and perceptual similarity network as a discriminator network. iPANs introduced new paired input conditions for the replacement of conditional adversarial networks to improve the image-to-image translation tasks. In this method the ground-truth images which are identical images are the real pair, whereas the generated images and ground-truth images are the fake pair.
- ID-CGAN [3]: ID-CGAN introduced to handle the image de-raining task by combining the pixel-wise least-squares reconstruction loss, conditional generative adversarial losses, and perceptual losses. ID-CGAN used cGAN structure to map from rainy images to de-rainy images. ID-CGAN consists of a dense generator to transform from an input image to its counter-part output images. ID-CGAN used the pre-trained VGG-16 network to calculate the perceptual losses between generated and ground-truth images.

#### 4.6.1. Comparison with Pix2Pix-cGAN, PAN, UTN-GAN and iPANs

**Figure 7:**
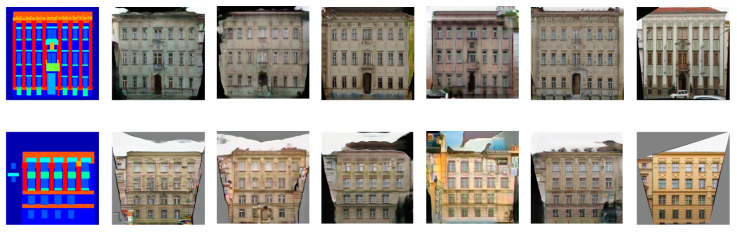

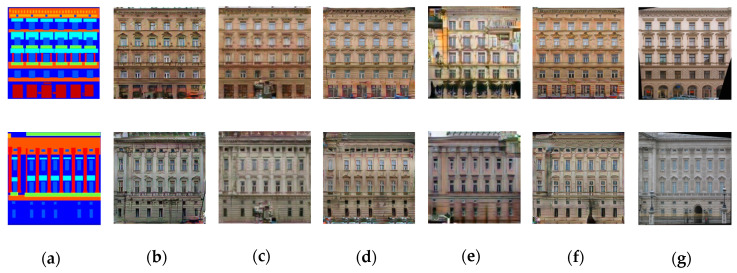
Samples results from paired labels to architectural photos. (**a**) input images, (**b**) results of pix2pix-cGAN, (**c**) results of UTN-GAN, (**d**) results of PAN, (**e**) results of iPANs, (**f**) results of the IP-RAN, and (**g**) targeted photos.

We attempt to transform semantic labels to architectural photos. This inverse conversion is a complicated process and distinct from the tasks of image segmentation. Pix2Pix-cGAN and UTN-GAN used adversarial and reconstruction losses, and PAN and iPANs used adversarial and perceptual losses to produce labels to architectural photos as shown in Figure 7. After the comparison, we observe the adopted approach captures further information and generates realistic and more similar images to the targeted photos with less deformation. Furthermore, the quantitative assessment in Table 4 also demonstrates that the IP-RAN can attain substantially improved results.

**Figure 8:**
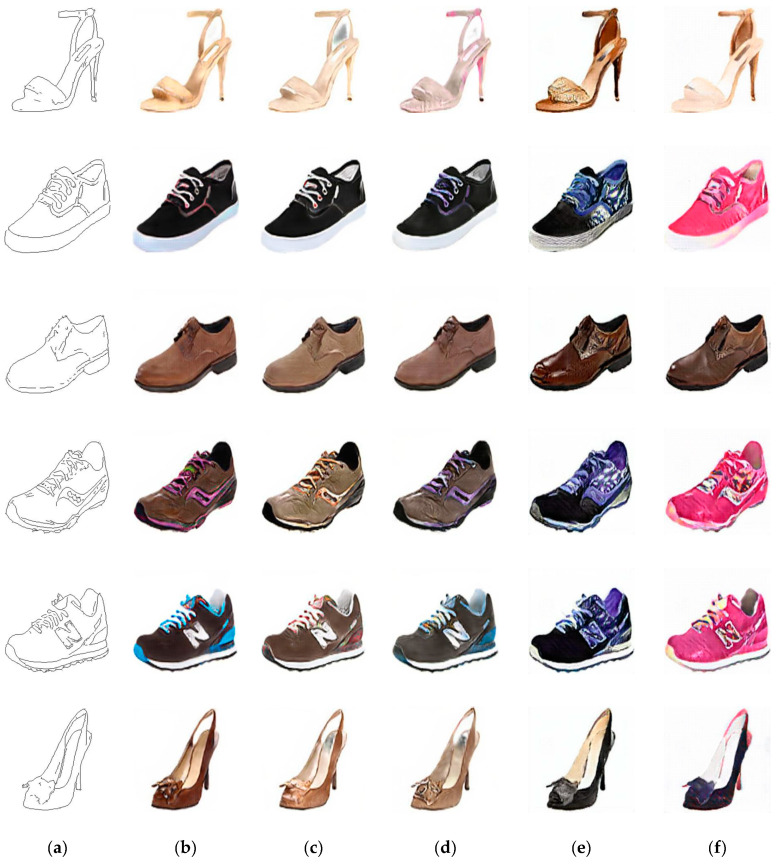
Samples result from edges to shoes. (**a**) input images, (**b**) results of the IP-RAN, (**c**) results of the pix2pix-cGAN, (**d**) results of PAN, (**e**) results of UTN-GAN, and (**f**) results of iPANs

Creating a real-world object from the corresponding input edges is one of the image-to-image conversion tasks as well. We train the IP-RAN on the dataset given by [23] to convert edges-to-shoes and compare its results by the outcomes of pix2pix-cGAN, PAN, UTN-GAN and iPANs. Figure 8 shows shoe photos generated from given input edges by the proposed method, pix2pix-cGAN, PAN, UTN-GAN and iPANs, while Table 5 presents the quantitative measures on the test set results. By observing and comparing the constructed shoe photos, we find that the IP-RAN, pix2pix-cGAN and PAN accomplished promising results, so far, it’s difficult to express which of these is better. On the measurement score of UQI and FID, the IP-RAN performed slightly weak compared to pix2pix-cGAN and PAN, yet superior in the other quantitative measures.

#### 4.6.2. Comparison with UTN-GAN, ID-CGAN and iPANs

ID-CGAN and iPANs try to resolve the image de-raining problem. They aim to eliminate rain streaks from a given input rainy photos. Assuming un-predictable weather situations, the image de-raining or de-snowing alone is a challenging image-to-image conversion problem.

**Figure 9:**
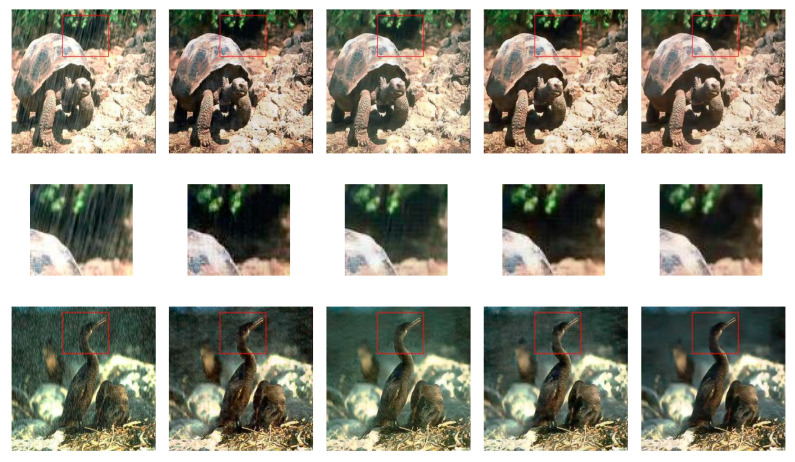

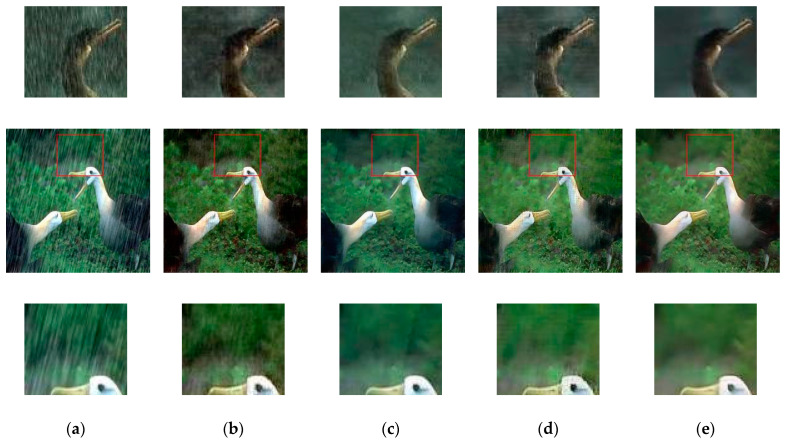
Sample results of synthetic test images. For good visual comparison, the smaller images below the test images represent specific regions-of-interest. (**a**) input images, (**b**) results of UTN-GAN, (**c**) results of ID-CGAN, (**d**) results of iPANs and (**e**) results of the IP-RAN.

**Figure 10:**
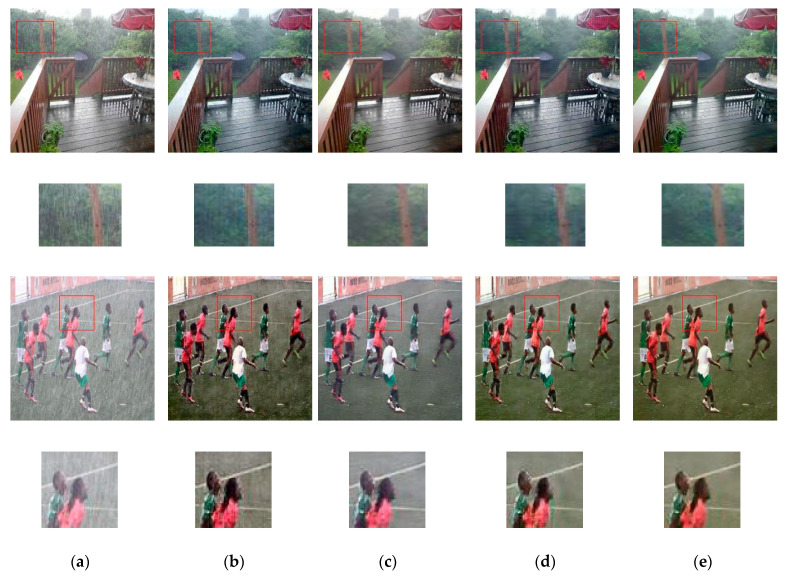
Sample results of real-world rainy images. For good visual comparison, the smaller images below the test images represent specific regions-of-interest. (**a**) input images, (**b**) results of UTN-GAN, (**c**) results of ID-CGAN, (**d**) results of iPANs and (**e**) results of the IP-RAN.

We try to resolve a single image de-raining task by the IP-RAN using a similar configuration to ID-CGAN. We train our adopted scheme on the image de-raining dataset provided by ID-CGAN [3]. This dataset contains 700 synthesizing images for training, whereas 100 artificial and 50 real-world rainy images are presented for testing purposes. Figure 9 shows the sample results of synthetic test images. As per the collection of ground-truth images are available against the set of synthetic test photos, we measure and report the quantitative outcomes in Table 6. Furthermore, we assess UTN-GAN, ID-CGAN, iPANs and IP-RAN on natural rainy images, and the results are shown in Figure 10.

From Figure 9 and Figure 10, we can observe that ID-CGAN, iPANs and the IP-RAN have accomplished great results in image de-raining tasks. The findings of the iPANs look slightly better, but contain some artifacts and blurriness. However, by examining the results carefully, the adopted scheme eliminates more rain-streaks with a lesser amount of color distortion. Moreover, as specified in Table 6, for a synthetic set of test images, the introduced method’s evaluation scores and the resultant images are far more comparable with the corresponding ground-truth photos than with the results of the other methods. In the single image de-raining problem, the adopted method can accomplish more improved results than UTN-GAN, ID-CGAN, iPANs; one of the possible reasons is that these methods used skip-connection in their generator network. These skip-connection passes useful as well as unwanted information directly from the input image to the output images throughout the network and influence the results. Even though ID-CGAN achieved highest score in the PSNR and UQI metrics, still rain-streak can be seen in the resultant images of ID-CGAN. On the other hand, the adopted method tries to resolve the problem through the proposed loss function using an encoder-decoder generator structure. The novel training scheme of IP-RAN can benefit the generator to learn better-quality mapping from the input images to the output images, leading to improved performance.

## 5. Conclusions

We have introduced a novel cGAN-based scheme to overcome the lack of information in input labels for imperfect paired datasets. In this work, we propose a novel Input-Perceptual and Reconstruction Adversarial Network (IP-RAN) for paired image-to-image conversion tasks as a general-purpose framework. We merge the input-perceptual loss with the adversarial and the per-pixel reconstruction Euclidean losses as an innovative loss function for imperfect paired datasets. Also, we analyze two popular generator configurations and evaluated their results quantitatively and qualitatively. A generator without skip-connections produced much better and visually pleasing results than a generator with skip-connections. We conducted extensive experiments on multiple datasets to assess the efficiency of the IP-RAN. The adopted scheme outperforms the state-of-the-art works for image-to-image conversion problems. The experimental results of several image-to-image conversion tasks illustrated that the proposed framework is efficient and capable of practical imperfect paired image-to-image conversion applications. In this study, we explored input-perceptual losses to feed the extra information of imperfect paired datasets for only paired image-to-image conversion tasks. Future work is required to examine the impact of input-perceptual losses for unpaired image-to-image conversion applications.

## Figures and Tables

**Table 1:** Comparison between state-of-the-art and proposed method.

Methods	Advantages	Disadvantages
CNNs (Reconstruction L1 and L2 losses) based methods [1,17]	Need less computation as one network is to trained.	Need big datasets to train
	Fast and easy to train	Produce blurry results
Simple GAN (Adversarial Loss) based methods [21,26]	Can be trained with small datasets	More computation than CNNs as two different networks to be trained
	Produces sharp and realistic images	GAN networks are difficult to train
		There is an image artifacts problem
Adversarial, reconstruction and perceptual losses with skip-connections in generator network based methods [3,4,23,25,29,63]	Achieve good quality results than CNNs and simple GAN by combining two loss functions	Skip-connections affect the quality of generated images by directly passing unwanted input information to the output of the network.
	Skip-connections in generator configuration reduce vanishing gradient problem	
Proposed method	This method adds extra information to the objective function to optimize the results.	Need to calculate input-perceptual losses which increase training time
	Use the Resnet bottleneck structure in the generator configuration to reduce the vanishing gradient problem.	
	Achieves excellent results visually and quantitatively	

**Table 2:** Generator Network of IP-RAN.

	Operation	Pre-Reflection Padding	Kernel Size	Stride	Non-Linearity	Feature Maps
Encoder entry 2	Convolution	3	7	1	ReLU	64
	Convolution		3	2	ReLU	128
	Convolution		3	2	ReLU	256
Residual Blocks	Residual block	1	3	1	ReLU	256
	Residual block	1	3	1	ReLU	256
	Residual block	1	3	1	ReLU	256
	Residual block	1	3	1	ReLU	256
	Residual block	1	3	1	ReLU	256
	Residual block	1	3	1	ReLU	256
	Residual block	1	3	1	ReLU	256
	Residual block	1	3	1	ReLU	256
	Residual block	1	3	1	ReLU	256
Decoder	Deconvolutional		3	2	ReLU	128
	Deconvolutional		3	2	ReLU	256
	Convolutional	3	7	1	Tanh	256

**Table 3:** Quantitative results compared with different loss functions.

	PSNR(dB)	SSIM	UQI	VIF	FID
L1	**13.43**	**0.2837**	**0.8186**	**0.0627**	176.74
cGAN	11.86	0.1996	0.7722	0.0399	111.00
L1+cGAN=CGAN	12.80	0.2399	0.8035	0.0480	113.53
IP-RAN	12.84	0.2426	0.8052	0.0488	**110.29**

**Table 4:** Quantitative results of labels to architectural photos, bold results show good scores.

	PSNR(dB)	SSIM	UQI	VIF	FID
Pix2Pix-cGAN	**13.37**	**0.2559**	**0.8195**	**0.0541**	113.53
UTN-GAN	12.78	0.2362	0.8016	0.0481	111.86
PAN	12.82	0.2370	0.8030	0.0477	112.47
iPANs	11.46	0.1765	0.7603	0.0382	140.70
IP-RAN	12.84	0.2426	0.8052	0.0488	**110.29**

**Table 5:** Quantitative results of Edges to Shoes, bold results show good scores.

	PSNR(dB)	SSIM	UQI	VIF	FID
Pix2Pix-cGAN	19.33	0.7569	**0.9220**	0.2092	**59.93**
UTN-GAN	15.41	0.6588	0.8255	0.1786	104.9
PAN	19.11	0.7389	0.9187	0.2034	62.13
iPANs	15.71	0.6671	0.8444	0.1778	117.1
IP-RAN	**19.42**	**0.7608**	0.9179	**0.2153**	62.15

**Table 6:** Quantitative results of image de-raining, bold results show good scores.

	PSNR(dB)	SSIM	UQI	VIF	FID
UTN-GAN	21.81	0.7325	0.9056	0.2939	127.4
ID-CGAN	**24.42**	0.8490	**0.9433**	0.3708	76.71
iPANs	22.44	0.7687	0.9252	0.3101	112.72
IP-RAN	23.69	**0.8518**	0.9412	**0.3740**	**75.90**
